# Comprehensive capsular, lipopolysaccharide, virulence, and antimicrobial resistance profiling of *Pasteurella multocida* isolated from buffaloes in Vietnam: First report of capsular type D and predominant L2 genotype

**DOI:** 10.14202/vetworld.2025.4069-4081

**Published:** 2025-12-27

**Authors:** Thai Van Nguyen, T. T Hang Trinh, Trong Van Nguyen, Dinh Ng–Nguyen, Hieu Quoc Nguyen, Hung Vu–Khac

**Affiliations:** 1 Faculty of Agriculture, Tay Nguyen University, Dak Lak, Vietnam; 2 Institute of Biotechnology and Environment, Nha Trang University, Nha Trang, Vietnam; 3 Department of Biotechnology, Institute of Veterinary Research and Development of Central Vietnam, Nha Trang, Vietnam

**Keywords:** antimicrobial resistance genes, buffalo, capsular typing, hemorrhagic septicemia, lipopolysaccharide genotype, molecular epidemiology, One Health, *Pasteurella multocida*, Vietnam, virulence–associated genes

## Abstract

**Background and Aim:**

*Pasteurella multocida* is a primary cause of hemorrhagic septicemia (HS) in buffaloes across tropical regions, leading to rapid deaths and significant economic losses. In Vietnam, recurrent HS outbreaks have been reported, yet most studies have solely focused on bacterial isolation or species-level identification. Comprehensive molecular data, including capsular typing, lipopolysaccharide (LPS) genotyping, virulence–associated genes (VAGs), and antimicrobial resistance genes (ARGs), remain limited for buffalo-derived strains. This molecular gap hampers understanding of strain diversity, epidemiology, and vaccine development. Notably, no previous Vietnamese study has concurrently characterized capsule, LPS, VAG, and ARG profiles or reported atypical serogroups in buffaloes. Therefore, integrated molecular surveillance is crucial to detect emerging lineages and guide One Health–oriented disease management. This study aimed to provide the first comprehensive molecular characterization of *P. multocida* strains isolated from buffaloes in Vietnam’s Central Highlands, incorporating capsular typing, LPS genotyping, virulence gene profiling, and antimicrobial resistance detection.

**Materials and Methods:**

Sixty-seven *P. multocida* isolates were recovered from lungs, bone marrow, and nasal swabs of clinically affected buffaloes (2022–2025). Species confirmation and molecular screening for capsular types (A, B, D), LPS genotypes (L1–L8), 12 VAGs, and seven ARGs were performed using polymerase chain reaction-based assays. Three representative isolates underwent 16S rRNA sequencing for phylogenetic analysis. Prevalence estimates, along with their 95% confidence intervals, were calculated, and chi-square tests were performed.

**Results:**

Capsular type B was the most common (62.7%), followed by type A (31.3%). Importantly, capsular type D (5.9%) was identified for the first time in Vietnamese buffaloes. LPS genotyping showed L2 as the predominant genotype (56.7%), with L6 (19.4%), L1 (16.4%), and L3 (7.5%) also present. All isolates contained eight conserved VAGs, while *pfhA* (58.2%) and *hgbB* (34.3%) showed variable presence. *ToxA* and *nanH* were not detected. Four ARGs, *floR* (22.3%), *tetB* (11.9%), *bla*ROB1 (10.4%), and *tetH* (4.4%), were observed. Phylogenetic analysis clustered all isolates within the *P. multocida* group, with the type D isolate forming a minor diverging sub-branch.

**Conclusion:**

This study establishes the first multilocus molecular profile of *P. multocida* in Vietnamese buffaloes, highlighting the emergence of capsular type D and the dominance of L2 genotypes. These findings support better diagnostics, vaccine antigen selection, and antimicrobial stewardship. Future work using multilocus sequence typing/whole–genome sequencing across livestock species will improve understanding of regional transmission dynamics within a One Health framework.

## INTRODUCTION

*Pasteurella multocida* is a Gram–negative bacterium responsible for a wide range of diseases in animals and humans, from mild infections to severe, often fatal conditions. These include hemorrhagic septicemia (HS) in cattle and buffaloes, pneumonic and septicemic pasteurellosis in sheep and goats, fowl cholera in poultry, and various infections in companion animals such as cats and dogs [1–4]. Among these diseases, HS is especially devastating in tropical regions, causing high morbidity and mortality. In Vietnam, recurrent HS outbreaks have been documented in both cattle and buffaloes, with buffaloes showing greater susceptibility and higher death rates. This highlights the need for focused molecular surveillance and targeted disease control strategies in this species.

Capsular typing, particularly the identification of serogroup B, remains essential for determining *P. multocida* strains involved in HS outbreaks across Asia [5–7]. Lipopolysaccharide (LPS) genotyping adds an extra layer of strain differentiation, as LPS types (L1–L8) display host- and disease-specific patterns; however, data on buffalo-derived strains, especially in Vietnam, are limited [8]. Virulence–associated genes (VAGs), which encode factors related to adhesion, iron acquisition, outer membrane stability, and enzymatic activity, further influence the pathogenic profile of *P. multocida* [1, 9]. Although the distribution of these genes varies among host species and regions, few studies have investigated VAGs patterns in Vietnamese buffalo isolates.

Antimicrobial resistance genes (ARGs) have been detected in *P. multocida* strains from pigs in Vietnam [10], but similar studies on buffalo-derived isolates are lacking. Although *P. multocida* has been isolated from various domestic animals, including cattle, buffaloes, pigs, poultry, goats, and sheep [11–13], most previous Vietnamese research focused only on species-level identification. Few studies have investigated capsular types, and almost none have assessed LPS genotypes or virulence profiles in buffalo strains. Consequently, essential molecular epidemiological data are still missing. Recent research across Asia emphasizes the importance of combining capsular and LPS genotyping to monitor emerging *P. multocida* lineages [14, 15]; however, similar molecular insights for Vietnamese buffalo populations are still unavailable.

Despite the frequent occurrence of HS in Vietnam and the recognized role of *P. multocida* as a major pathogen in buffaloes, there is still a significant lack of molecular epidemiological data on the circulating strains in this host. Previous Vietnamese studies have mainly focused on bacterial isolation and species confirmation, with limited work on capsular typing and almost no research combining LPS genotyping, virulence–associated gene (VAG) profiles, and ARG patterns in buffalo-derived isolates. Consequently, critical information about strain diversity, pathogenic potential, and the molecular factors that influence disease severity in buffalo populations is missing. Importantly, buffaloes in the Central Highlands are raised within multispecies livestock systems, often near cattle, pigs, poultry, and dogs, which heightens the risk of cross-species transmission, the emergence of unusual capsular or LPS types, and the spread of ARG. While recent Asian studies highlight the importance of combined capsular–LPS genotyping for tracking new *P. multocida* lineages, comparable integrated data for Vietnamese buffaloes are still lacking. This significant gap hampers the development of targeted vaccines, accurate diagnostic tools, and effective regional control strategies.

To address these gaps, this study aimed to perform the first comprehensive molecular characterization of *P. multocida* isolated from buffaloes in Vietnam’s Central Highlands. Using a PCR-based multilocus approach, we examined capsular types, LPS genotypes, and VAG profiles, along with screening for key antimicrobial resistance determinants. Additionally, representative isolates underwent 16S rRNA sequencing to confirm species identity and determine phylogenetic relationships with global reference strains. By integrating capsule, LPS, virulence, and resistance markers, this research offers a comprehensive molecular epidemiological assessment of *P. multocida* in Vietnamese buffalo populations. The data generated aims to improve diagnostic accuracy, guide vaccine antigen selection based on regional strain patterns, strengthen antimicrobial stewardship, and support future One Health surveillance efforts in mixed-species farming settings.

## MATERIALS AND METHODS

### Ethical approval

This study did not involve any experimental procedures on live animals. All samples used in this research were collected from buffaloes that were either clinically affected by HS or had died naturally and were submitted to provincial veterinary authorities for routine diagnostic investigation. No animals were handled, restrained, or sampled for research purposes. Accordingly, the work did not require a separate Institutional Animal Care and Use Committee approval under the ethical policies of Tay Nguyen University and the Institute of Veterinary Research and Development of Central Vietnam.

Sampling and laboratory procedures complied with the national regulations of Vietnam on animal welfare, biosafety, and disease surveillance, as well as guidelines outlined by the World Organization for Animal Health (OIE/WOAH) for the humane use of animals in research and diagnosis. All submitted samples were processed anonymously, and no identifying information related to farms or owners was disclosed.

Owner consent was obtained at the time of sample submission for diagnostic purposes, including permission to use leftover clinical materials for research aimed at improving disease control in the region. All laboratory analyses, bacterial isolation, polymerase chain reaction (PCR) assays, virulence and resistance gene screening, and sequencing, were performed on stored isolates without requiring any interaction with live animals. Thus, the study fully adhered to the principles of Replacement, Reduction, and Refinement (3Rs) in animal research.

### Study period and location

**Figure 1:**
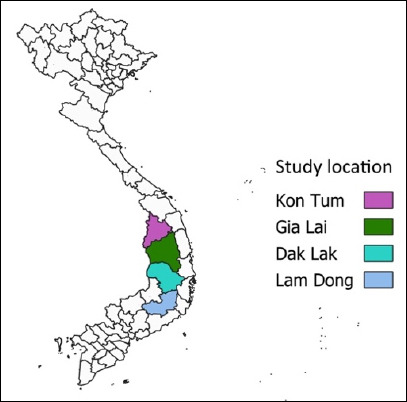
Map of Vietnam showing the study location.

The study was conducted from April 2022 to May 2025. All samples were collected from buffaloes raised in the Dak Lak, Gia Lai, Kon Tum, and Lam Dong provinces of the Central Highlands in Vietnam. Sampling took place over a three-year span (Figure 1).

### Isolation and identification of *P. multocida* strains

A total of 67 *P. multocida* isolates were obtained from the lungs, bone marrow, and nasal swabs of buffaloes showing clinical signs of pasteurellosis, such as fever, edema of the mandible, neck, and brisket, nasal discharge, respiratory distress, and loss of appetite. All isolates were cultured on 5% sheep blood agar (Oxoid, UK) and incubated at 37°C for 24 h. Initial identification was based on characteristic morphology (smooth, grayish-white, non-hemolytic colonies), Gram staining, absence of growth on MacConkey agar (HiMedia, India), and standard biochemical profiles (positive catalase and oxidase tests; fermentation of glucose and mannitol). Species confirmation was carried out using a *P. multocida*–specific PCR assay with ATCC 12945 (Microbiologics, USA) as the positive-control, following the previously described method [16].

### DNA extraction

Confirmed isolates were cultured in brain–heart infusion broth (Oxoid, UK) at 37°C for 24 h. A 1 mL aliquot of the overnight culture was processed for DNA extraction using the QIAamp DNA Mini Kit (Qiagen, Germany) following the manufacturer’s instructions. Extracted DNA was stored at –20°C until analysis.

### Capsular typing

Capsular types were identified using multiplex PCR targeting the *capA*, *capB*, and *capD* loci [17, 18]. Each 20 μL reaction contained 2 μL of DNA template, 10 μL of 2× MyTaq Mix (Bioline, USA), 0.5 μL of each primer (25 μM; Phusa Genomics, Vietnam), and nuclease-free water. The thermal cycle included 94°C for 4 min; 35 cycles of 94°C for 45 s, 55°C for 45 s, and 72°C for 45 s; followed by a final extension at 72°C for 10 min. Positive-control DNA provided by the Institute of Veterinary Research and Development of Central Vietnam (IVRD) was included in each run.

### LPS genotyping

LPS genotypes were identified using two multiplex PCR sets targeting eight LPS-related genes, following a previously described method with minor modifications [8]. In the original protocol, eight LPS-specific genes were amplified in a single multiplex reaction; in the present study, these eight targets were divided into two separate four-plex multiplex PCR assays:

- Multiplex set 1: *pcgD–pcgB, gatF, latB, ppgB*
- Multiplex set 2: *nctA, rmlA–rmlC, nctB, natG*

Each 50 μL reaction included 25 μL of MyTaq Mix, 0.5 μL of each primer (25 μM), 5 μL of DNA template, and 18 μL of nuclease-free water. Cycling conditions consisted of 96°C for 5 min; 30 cycles of 96°C for 30 s, 56°C for 30 s, and 72°C for 2.5 min; and a final extension at 72°C for 8 min. IVRD positive-control DNA was used to verify assay performance.

### Detection of VAGs

Twelve VAGs related to adhesion, iron acquisition, outer membrane integrity, and enzymatic activity were screened using multiplex PCR, following protocols adapted from previous studies [19–21].

- Multiplex set 1: *sodA, hgbA, ptfA, pfhA*
- Multiplex set 2: *ompH, tonB, nanH, toxA*
- Multiplex set 3: *oma87, sodC, hgbB, nanB*

Each 25 μL reaction contained 2 μL DNA template, 12.5 μL 2× MyTaq Mix, 0.5 μL of each primer (10 μM), and 6.5 μL nuclease-free water. Amplification conditions were: 96°C for 5 min; 30 cycles of 96°C for 60 s, 56°C for 60 s, and 72°C for 60 s; and a final extension at 72°C for 10 min. IVRD positive-control DNA was used for quality assurance. Primer sequences for all assays are listed in Table 1 [8, 16, 17, 20, 21, 22].

**Table 1:** Primers used for the identification, detection of capsular serotypes, lipopolysaccharide genotypes, virulence–associated genes, and *16S rRNA* of *Pasteurella multocida.*

Gene	Function	Primer sequences (5’–3’)	Amplicon size (bp)	References
*kmt1*	Species–specific marker for *P. multocida*	F: ATCCGCTATTTACCCAGTGG R: GCTGTAAACGAACTCGCCAC	460	[16]
*hyaD-hyaC*	Serogroup A cap	F: GATGCCAAAATCGCAGTCAG R: TGTTGCCATCATTGTCAGTG	1044	[17]
*bcbD*	Serogroup B cap	F: CATTTATCCAAGCTCCACC R: GCCCGAGAGTTTCAATCC	760	[17]
*dcf*	Serogroup D cap	F: TTACAAAAGAAAGACTAGGAGCCC R: CATCTACCCACTCAACCATATCAG	657	[17]
*pcgD–pcgB*	LPS genotype L1	F: ACATTCCAGATAATACACCCG R: ATTGGAGCACCTAGTAACCC	1307	[8]
*nctA*	LPS genotype L2	F: CTTAAAGTAACACTCGCTATTGC R: TTTGATTTCCCTTGGGATAGC	810	[8]
*gatF*	LPS genotype L3	F: TGCAGGCGAGAGTTGATAAACCATC R: CAAAGATTGGTTCCAAATCTGAATGGA	474	[8]
*latB*	LPS genotype L4	F: TTTCCATAGATTAGCAATGCCG R: CTTTATTTGGTCTTTATATATACC	550	[8]
*rmlA–rmlC*	LPS genotype L5	F: AGATTGCATGGCGAAATGGC R: CAATCCTCGTAAGACCCCC	1175	[8]
*nctB*	LPS genotype L6	F: TCTTTATAATTATACTCTCCCAAGG R: AATGAAGGTTTAAAAGAGATAGCTGGAG	668	[8]
*ppgB*	LPS genotype L7	F: CCTATATTTATATCTCCTCCCC R: CTAATATATAAACCATCCAACGC	931	[8]
*natG*	LPS genotype L8	F: GAGAGTTACAAAAATGATCGGC R: TCCTGGTTCATATATAGGTAGG	255	[8]
*ptfA*	Type IV fimbria (adhesion)	F: TGTGGAATTCAGCATTTTAGTGTGTC R: TCATGAATTCTTATGCGCAAAATCCTGCTGG	488	[20]
*pfhA*	Filamentous hemagglutinin (adhesion)	F: AGCTGATCAAGTGGTGAAC R: TGGTACATTGGTGAATGCTG	275	[21]
*nanB*	Sialidase	F: GTCCTATAAAGTGACGCCGA R: ACAGCAAAGGAAGACTGTCC	554	[21]
*nanH*	Sialidase	F: GAATATTTGGGCGGCAACA R: TTCTCGCCCTGTCATCACT	360	[21]
*ompH*	Porin (protectin)	F: CGCGTATGAAGGTTTAGGT R: TTTAGATTGTGCGTAGTCAAC	438	[21]
*oma87*	Porin (protectin)	F: ATGAAAAAACTTTTAATTGCGAGC R: TGACTTGCGCAGTTGCATAAC	948	[21]
*exbB–tonB*	Iron metabolism	F: GGTGGTGATATTGATGCGGC R: GCATCATGCGTGCACGGTT	1144	[21]
*hgbA*	Hemoglobin-binding protein (iron uptake)	F: TGGCGGATAGTCATCAAG R: CCAAAGAACCACTACCCA	419	[21]
*hgbB*	Hemoglobin-binding protein (iron uptake)	F: ACCGCGTTGGAATTATGATTG R: CATTGAGTACGGCTTGACAT	788	[21]
*toxA*	Dermonecrotic toxin	F: CTTAGATGAGCGACAAGGTT R: GGAATGCCACACCTCTATA	865	[21]
*sodA*	Superoxide dismutate	F: TACCAGAATTAGGCTACGC R: GAAACGGGTTGCTGCCGCT	361	[21]
*sodC*	Superoxide dismutate	F: AGTTAGTAGCGGGGTTGGCA R: TGGTGCTGGGTGATCATCATG	235	[21]
*16S rRNA*	Universal bacterial marker	F: AGAGTTTGATYMTGGC R: GYTACCTTGTTACGACTT	1502	[22]

PCR = Polymerase chain reaction

### Detection of ARGs

ARGs were screened using simplex PCR with primers listed in Table 2 [23–25]. Each 25 μL reaction included 2 μL of DNA template, 12.5 μL of 2× MyTaq Mix, 0.5 μL of each primer (10 μM), and 9.5 μL of water. Cycling conditions were the same as those used for 16S rRNA PCR, except for *tetO*, where the extension step was increased to 1 min [10]. Positive-control DNA from IVRD was included in all runs.

**Table 2:** Primer pairs and polymerase chain reaction products for the detection of antimicrobial resistance genes.

Antibiotic class	Gene	Primer sequences (5’–3’)	Amplicon size (bp)	References
β-lactam	*blaTEM*	F: GAGTATTCAACATTTTCGT R: ACCAATGCTTAATCAGTGA	852	[23]
	*blaROB1*	F: CATTAACGGCTTGTTCGC R: CTTGCTTTGCTGCATCTTC	856	[23]
Tetracycline	*tetB*	F: CCTTATCATGCCAGTCTTGC R: ACTGCCGTTTTTTTCGCC	774	[23]
	*tetH*	F: ATACTGCTGATCACCGT R: TCCCAATAAGCGACGCT	1076	[23]
	*tetO*	F: TAACTTAGGCATTCTGGCTC R: TCAAGCAGACTCCCTGCCCATTTGT	1801	[23]
Chloramphenicol	*floR*	F: CACGTTGAGCCTCTATATGG R: ATGCAGAAGTAGAACGCGAC	885	[24]
Aminoglycoside (Gentamycin)	*aacA4*	F: CTCGAATGCCTGGCGTGTTT R: TTGCGATGCTCTATGAGTGGCTA	482	[25]

### *16S rRNA* gene sequencing and phylogenetic analysis

Three isolates representing capsular types A, B, and D and harboring multiple VAGs were selected for 16S rRNA sequencing using universal primers [22]. PCR reactions (25 μL) contained 2 μL of DNA template, 0.5 μL of each primer (10 μM), 12.5 μL of 2× MyTaq Mix, and 9.5 μL of water. Conditions were: 94°C for 5 min; 30 cycles at 94°C for 45 s, 48°C for 45 s, and 72°C for 90 s; and a final extension at 72°C for 8 min.

Amplicons were sequenced through Sanger sequencing (1st BASE, Singapore). Sequences were aligned in BioEdit (version 5.0.9) using the ClustalW algorithm. Phylogenetic trees were generated in MEGA version 6.06 (Molecular Evolutionary Genetics Analysis; www.megasoftware.net) with the neighbor-joining method, performing 1,000 bootstrap replicates; bootstrap values ≥70% indicated strong support. Rooting was carried out using *P. pneumotropica* NCTC 8141 (AF362924).

All PCR assays were conducted in duplicate on a C1000 Touch thermal cycler (Bio-Rad, USA). PCR products were separated on 1% TBE agarose gels at 130 V for 60 min, stained with ethidium bromide, and visualized under UV light using a 100-bp DNA ladder (Bioline, USA).

### Statistical analysis

Prevalence was calculated as the proportion of positive isolates among total samples and expressed as percentages with 95% confidence intervals (R version 4.4.3, R Core Team, Vienna, Austria). Differences between proportions were assessed using chi-square tests, with p < 0.05 considered statistically significant. QGIS version 3.10.0 was used to generate the study site map [26].

## RESULTS

### Identification and confirmation of P. multocida

**Figure 2:**
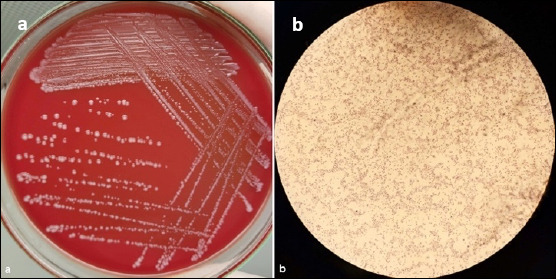
Morphological characteristics of *Pasteurella multocida* isolates. (a) Colonies of *P. multocida* after 24 h incubation at 37^o^C on 5% sheep blood agar showing smooth, glistening, non–hemolytic morphology. (b) Gram–strained smear demonstrating small Gram–negative coccibacilli observed under oil immersion (100×).

**Figure 3:**
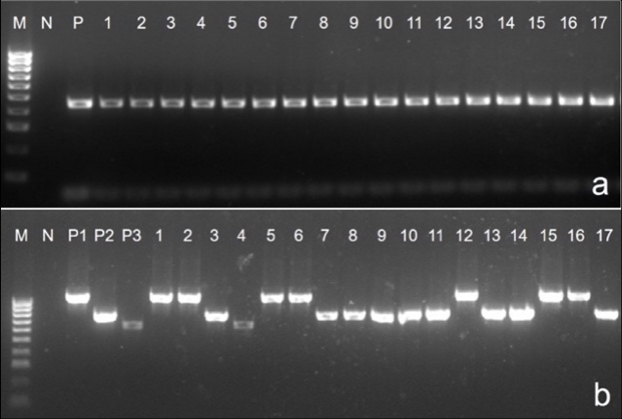
Species–specific Polymerase chain reaction (PCR) and multiplex PCR for capsular typing of *Pasteurella multocida*. (a) *P. multocida* confirmation by species–specific PCR. 1-17: representative samples; N: negative control; P: positive-control (460 bp); M: 100 bp DNA ladder. (b) Multiplex reaction of *capA*, *capB*, *capD*. 1-17: representative samples; P1: *capA* positive-control (1044 bp); P2: *capB* positive-control (760 bp); P3: *capD* positive-control (657 bp); M: 100 bp DNA ladder.

Biochemical characterization confirmed that all 67 isolates showed typical features of *P. multocida*, including Gram–negative coccobacilli morphology (Figures 2a and 2b), lack of growth on MacConkey agar, non-hemolytic colonies on blood agar, and positive catalase and oxidase reactions. Glucose and sucrose fermentation were observed, while lactose was not fermented. Species–specific PCR further verified that all isolates belonged to *P. multocida* (Figure 3a).

### Distribution of capsular biosynthesis genes

Multiplex PCR analysis revealed the following capsular gene distribution (Table 3; Figure 3b):

**Table 3:** The distribution of capsular biosynthesis genes of *Pasteurella multocida* detected by polymerase chain reaction (n = 67).

Gene	No. of positive isolates	Percentage	95% CI
*hyaD-hyaC (capA)*	21	31.3^a^	20.5–43.8
*bcbD (capB)*	42	62.7^b^	50.1–74.2
*dcdF (capD)*	4	5.9^c^	1.7–14.5

Different letters in a column denote a significant difference (p < 0.05), CI = Confidence interval

- *capA*: 21 isolates (31.3%)
- *capB*: 42 isolates (62.7%)
- *capD*: 4 isolates (5.9%)

Thus, capsular type B predominated among buffalo-derived isolates, followed by types A and D.

### LPS genotype distribution

**Figure 4:**
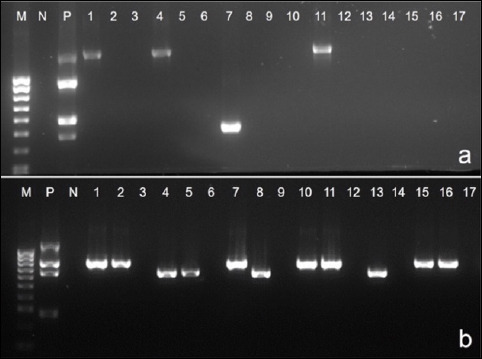
Polymerase chain reaction (PCR) for lipopolysaccharide genotypes of isolated *Pasteurella multocida* strains. 1-17: samples, N: negative control, P: positive-control, M: 100 bp DNA ladder. (a) PCR product sizes: L1 = 1307 bp; L3 = 474 bp. (b) PCR product sizes: L2 = 810 bp; L6 = 668 bp.

Four LPS genotypes were detected among the 67 isolates (Table 4; Figures 4a and 4b):

**Table 4:** Distribution of lipopolysaccharides genotypes among *Pasteurella multocida* isolates (n = 67).

Gene	No. of positive isolates	Percentage (%)	95% CI
*pcgD–pcgB (L1)*	11	16.4^a^	8.4-27.5
*nctA (L2)*	38	56.7^b^	44.1-68.7
*gatF (L3)*	5	7.5^a^	2.4-16.5
*latB (L4)*	0	–	–
*rmlA–rmlC (L5)*	0	–	–
*nctB (L6)*	13	19.4^a^	10.7-30.8
*ppgB (L7)*	0	–	–
*natF (L8)*	0	–	–

Different letters in a column denote a significant difference (p < 0.05), CI = Confidence interval

- L2: 38 isolates (56.7%) - most prevalent)
- L6: 13 isolates (19.4%)
- L1: 11 isolates (16.4%)
- L3: 5 isolates (7.5%)

Genotypes L4, L5, L7, and L8 were not detected in any isolate.

### VAGs

All isolates carried eight conserved VAGs: *ptfA*, *nanB*, *ompH*, *oma87*, *exbB–tonB*, *hgbA*, *sodA*, and *sodC*. Variable gene frequencies included:

- *pfhA*: 58.2%
- *hgbB*: 34.3%

**Figure 5:**
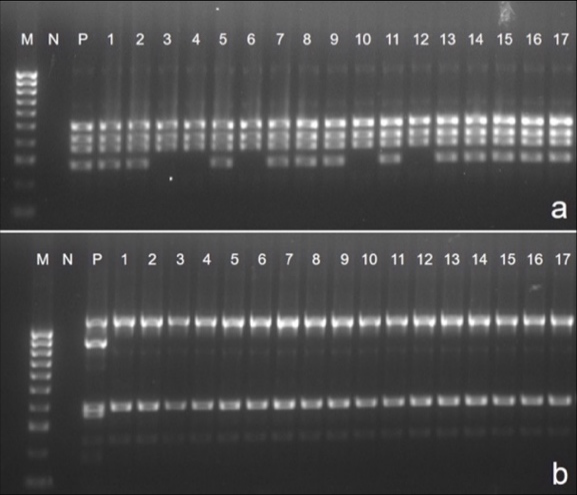
Multiplex polymerase chain reaction (PCR) detection of virulence–associated genes in *Pasteurella multocida* isolates. M: 100 bp DNA ladder, N: negative control, P: positive-control, 1-17: samples. (a) PCR product sizes: *ptfA* = 488 bp; *hgbA* = 419 bp; *sodA* = 361 bp; *pfhA* = 275 bp. (b) PCR product sizes: *tonB* = 1144 bp; *ompH* = 438 bp.

The *nanH* and *toxA* genes were completely absent across all isolates. Representative PCR amplifications are shown in Figures 5a and 5b, with detailed prevalence in Table 5.

**Table 5:** Prevalence of virulence–associated genes in *Pasteurella multocida* isolates (n = 67).

Gene	No. of positive isolates	Percentage (%)	95% CI
*ptfA*	67	100^a^	94.6-100
*pfhA*	39	58.2^b^	45.5-70.1
*nanB*	67	100^a^	94.6-100
*nanH*	0	-	-
*ompH*	67	100^a^	94.6-100
*oma87*	67	100^a^	94.6-100
*exbB–tonB*	67	100^a^	94.6-100
*hgbA*	67	100^a^	94.6-100
*hgbB*	23	34.3^c^	23.1-46.9
*toxA*	0	-	-
*sodA*	67	100^a^	94.6-100
*sodC*	67	100^a^	94.6-100

Different letters in a column denote a significant difference (p < 0.05), CI = Confidence interval

### Phylogenetic analysis of 16S rRNA sequences

**Figure 6:**
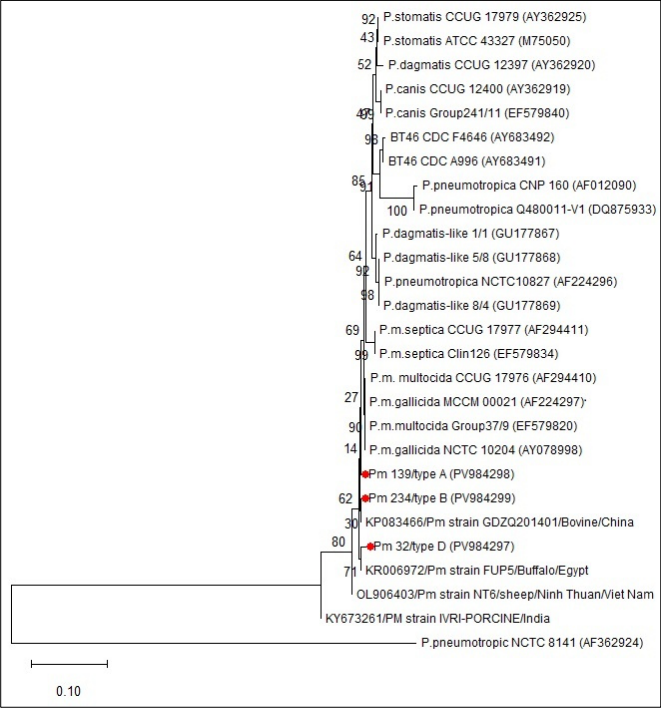
Neighbor-joining dendrogram illustrating the phylogenetic relationships of *16S rRNA* gene sequences of *Pasteurella multocida* isolates.

Phylogenetic analysis showed that isolates Pm 139 (type A), Pm 234 (type B), and Pm 32 (type D) clearly grouped within the *P. multocida* species, confirming their identity (Figure 6).

- Pm 139/type A and Pm 234/type B grouped closely with *P. multocida* reference strains (e.g., NCTC 10204), indicating high genetic similarity.
- Pm 32/type D formed a distinct but related sub-branch, suggesting minor genetic divergence while remaining within the species cluster.

All three isolates were distinctly separated from other *Pasteurella* species (*Pasteurella stomatis*, *Pasteurella dagmatis*, *Pasteurella canis*, and *Pasteurella pneumotropica*). International reference strains from China, Egypt, India, and Vietnam also grouped within the *P. multocida* cluster, showing conservation of 16S rRNA sequences across different geographic regions.

The neighbor-joining tree was built using 1,000 bootstrap replications, with bootstrap values of 70% or higher considered strong support *P. pneumotropica* NCTC 8141 (AF362924) served as the outgroup.

### Occurrence of ARGs

**Figure 7:**
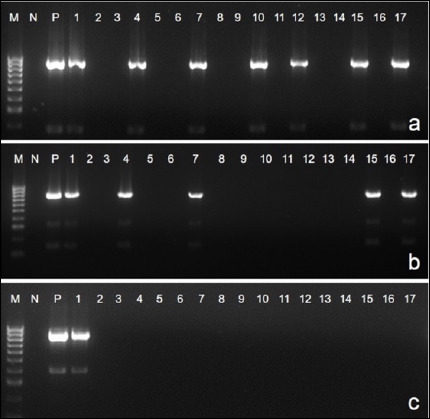
Polymerase chain reaction assays for detecting the prevalence of (a) *floR*, (b) *tetB*, and (c) *blaROB1*.

ARG detection revealed variable resistance gene prevalence (Table 6 and Figures 7a–c):

**Table 6:** The distribution of virulence–associated genes in *Pasteurella multocida* (n = 67).

Gene	No. of positive isolates	Percentage (%)	95% CI
*blaTEM*	0	-	-
*blaROB1*	7	10.4^ab^	4.3-20.3
*tetB*	8	11.9^ab^	5.2-22.1
*tetH*	3	4.4^a^	0.9-12.5
*tetO*	0	-	-
*floR*	15	22.3^b^	13.1-34.2
*aacA4*	0	-	-

Different letters in a column denote a significant difference, CI = Confidence interval

*floR*: 22.3% (15/67) - highest prevalence
*tetB*: 11.9% (8/67)
*blaROB1*: 10.4% (7/67)
*tetH*: 4.4% (3/67)

The *blaTEM*, *tet*O, and *aac*A4 genes were not detected in any isolate.

## DISCUSSION

### Overall significance of the study

This investigation offers the first in-depth molecular characterization of *P. multocida* isolates from buffaloes in the Central Highlands of Vietnam. Previous studies in Vietnam mainly focused on species-level identification, leaving significant gaps in knowledge about capsular types, LPS genotypes, virulence–associated genes (VAGs), and ARGs. By incorporating these molecular markers, the current study provides new epidemiological insights and creates a baseline dataset for regional surveillance and disease management.

### Capsular type distribution and epidemiological implications

Capsular type B was the most common serogroup identified, aligning with reports from other Asian countries where serogroup B is strongly linked to HS in cattle and buffaloes [7, 14, 27]. Capsular type A was also found, consistent with findings from different geographic regions [27–30].

Importantly, the detection of capsular type D (5.9%; 4/67) in Vietnamese buffaloes is a new and epidemiologically important discovery, as this serogroup has not been previously reported in buffalo-derived isolates in Vietnam. Although uncommon, type D has been found in buffaloes in Iran (1.8%) [27] and India (4.3%) [29], indicating its sporadic yet widespread presence across Asia.

The appearance of type D in this study may be due to pathogen spillover within the multispecies farming systems typical of the Central Highlands, where buffaloes, cattle, pigs, poultry, and dogs are raised closely together [31]. Such interfaces promote interspecies transmission, genetic exchange, and the introduction of unusual serogroups. This finding underscores One Health concerns regarding the circulation of various *P. multocida* lineages and emphasizes the need for expanded molecular surveillance.

The dominance of capsular type B and its alignment with region-specific patterns highlight its significance as a target for better vaccine development in Vietnam.

### LPS genotype variation and ecological drivers

LPS genotype L2 was the most common, followed by L6, L1, and L3. This distribution is different from patterns typically seen in cattle, pigs, and poultry, where L3 and L6 are mostly found [32–34]. For example:

- L6 predominates in pig-derived isolates in China [33].
- L3 is common in bovine respiratory pasteurellosis [32].

The prominence of L2 in buffaloes may reflect host-specific ecology, antibiotic use, and local husbandry practices. In the Central Highlands, buffaloes often graze communally under extensive or semi-intensive systems, which promotes the transmission of hemorrhagic septicemia–associated strains. Seasonal humidity and flooding may also help *P. multocida* persist in the environment. These ecological factors probably contribute to the co-occurrence of capsular type B and L2, the dominant profile observed in this study.

### Virulence gene profiles and their biological relevance

All isolates carried conserved VAGs responsible for adhesion (*ptfA*), iron acquisition (*exbB–tonB* and *hgbA*), and outer membrane integrity (*omph* and *oma87*). The universal presence of *ptfA* and *nanB* is consistent with their known importance in colonization and mucosal invasion, mirroring reports from India [29] and Iran [35].

The moderate prevalence of *pfhA* (58.2%) and *hgbB* (34.3%) aligns with previous findings that the distribution of virulence genes varies by capsular type. Serogroup B isolates, which were predominant here, are historically linked to extensive adhesion and iron uptake capabilities [30]. The absence of *toxA* and *nanH* (0%) aligns with the host specificity of these toxin-related genes, which are more commonly associated with disease syndromes in pigs and poultry than in ruminants [21, 29].

These patterns support the idea that capsule genotype influences virulence gene composition and reflect a virulence profile adapted to buffalo-related HS.

### ARG patterns and One Health context

Among the seven ARGs screened, four, *blaROB1*, *tetB*, *tetH*, and *floR*, were detected at different frequencies, while *blaTEM*, *tetO*, and *aacA4* were not present. This is the first report of ARG profiles in buffalo-derived *P. multocida* isolates from Vietnam.

The overall ARG prevalence was significantly lower than that observed in Vietnamese pig isolates, in which all seven genes have been documented [10]. This difference probably stems from species–specific antimicrobial exposure and treatment strategies, highlighting the need for host-targeted antimicrobial stewardship.

International comparisons reveal substantial variation. For instance:

- Multidrug-resistant isolates in Egypt commonly carry *tetH* and β-lactamase genes [36].
- In Germany, *floR* was found in only 12.5% [37], which is less than the 22.3% observed in this study.

In Vietnam’s mixed-farming systems, close animal proximity heightens the risk of horizontal ARG gene transfer between species. Detection of *tetB*, *tetH*, and *blaROB1* matches the common field use of tetracyclines and β-lactams, while *floR* aligns with routine florfenicol administration [10, 38]. These findings highlight the importance of integrated molecular surveillance and careful antimicrobial use across livestock sectors within a One Health approach.

### Phylogenetic insights and evolutionary considerations

Phylogenetic analysis confirmed that all isolates clustered closely with global *P. multocida* reference strains from cattle, buffaloes, pigs, and sheep, indicating strong species-level genetic conservation. However, the capsular type D isolate (Pm 32) formed a distinct minor sub-branch, suggesting possible local microevolution within the multispecies farming ecosystems of the Central Highlands. These ecological interfaces may facilitate interspecies transmission, genetic recombination, and the emergence of new sub-lineages. Therefore, ongoing genomic surveillance, including MLST and whole–genome sequencing, is crucial to monitor evolutionary trends and transmission pathways.

## CONCLUSION

This study offers the first comprehensive molecular characterization of *P. multocida* isolated from buffaloes in the Central Highlands of Vietnam, providing important insights into the pathogen’s capsular composition, LPS diversity, virulence factors, antimicrobial resistance patterns, and phylogenetic placement. Among the 67 isolates examined, capsular type B was most common (62.7%), followed by type A (31.3%). Notably, capsular type D (5.9%) was identified for the first time in Vietnamese buffaloes, an epidemiologically important discovery indicating possible spillover within multispecies farming systems. LPS genotyping showed L2 as the dominant genotype (56.7%), with all isolates carrying eight conserved virulence genes (*ptfA*, *nanB*, *ompH*, *oma87*, *exbB–tonB*, *hgbA*, *sodA*, and *sodC*), and variable presence of *pfhA* (58.2%) and *hgbB* (34.3%). Four antimicrobial resistance genes, *floR*, *tetB*, *blaROB1*, and *tetH*, were detected, marking the first report of ARGs in buffalo-derived *P. multocida* in Vietnam.

The predominance of capsular type B and L2 LPS genotype has direct implications for HS vaccine development, as including regional dominant antigens may improve immunoprotection. The presence of specific virulence and resistance genes emphasizes the need for cautious antimicrobial use and the development of diagnostic panels tailored to buffalo-associated strains. The detection of capsular type D highlights the importance of monitoring cross-species pathogen transmission in mixed-farming systems.

This work employs multiple molecular methods, capsular typing, LPS genotyping, VAG profiling, ARG screening, and phylogenetic analysis, offering a multilayered epidemiological overview not previously available in Vietnam. The use of control strains, standardized PCR assays, and sequencing enhances the accuracy of the data. However, the study did not include phenotypic antimicrobial susceptibility testing, limiting direct correlation between ARGs and clinical resistance. Additionally, sampling was restricted to four provinces in the Central Highlands, and whole–genome sequencing was not conducted, which limits deeper insights into evolutionary pathways and transmission dynamics. Future research involving MLST and whole–genome sequencing across buffalo, cattle, pigs, and poultry will help clarify strain relationships, transmission routes, and the emergence of new lineages. Incorporating phenotypic resistance testing and environmental sampling could strengthen One Health surveillance strategies.

Overall, this study provides essential baseline molecular data for *P. multocida* in Vietnamese buffaloes and underscores the need for ongoing surveillance, targeted vaccination, and responsible antimicrobial use. The findings significantly contribute to regional disease management efforts and lay the groundwork for advanced genomic studies in Vietnam’s livestock sector.

## DATA AVAILABILITY

The 16S *rRNA* gene sequences generated in this study have been deposited in GenBank under the accession numbers PV984297, PV984298, and PV984299. Supplementary Table 1. Molecular characteristics (capsular type, LPS genotype, VAGs, and ARGs) of all *P. multocida* isolates included in this study.

## AUTHORS’ CONTRIBUTIONS

TVN: Performed the experiments, analyzed and interpreted the data, and, with support from TVN, TTHT, and HVK: Analyzed and interpreted the data, drafted and revised the manuscript. HVK: Conceived the study, provided project supervision, and critically revised the manuscript. TVN, DNN, and HQN: Collected the samples and contributed to data acquisition and interpretation. All authors have read and approved the final version of the manuscript.
